# Effect of the Supporting Electrolyte on Chloroform Reduction at a Silver Electrode in Aqueous Solutions

**DOI:** 10.3390/molecules26030525

**Published:** 2021-01-20

**Authors:** Anna M. Brudzisz, Agnieszka Brzózka, Grzegorz D. Sulka

**Affiliations:** 1 Faculty of Advanced Technology and Chemistry, Institute of Materials Science and Engineering, Military University of Technology, Kaliskiego 2, 00908 Warszawa, Poland; anna.brudzisz@wat.edu.pl; 2 Department of Physical Chemistry and Electrochemistry, Faculty of Chemistry, Jagiellonian University, Gronostajowa 2, 30387 Krakow, Poland; brzozka@chemia.uj.edu.pl

**Keywords:** silver electrode, supporting electrolyte, electrocatalytic reduction, trichloromethane

## Abstract

Herein, we report, for the first time, a comparative study on the electrocatalytic reduction of chloroform on silver in different aqueous supporting electrolytes. Cyclic voltammetry measurements were performed at a wide range of scan rates and concentrations of CHCl_3_ using 0.05 M NaClO_4_, NaH_2_PO4, and Na_2_HPO_4_ as supporting electrolytes. We observed that a type of supporting electrolyte anion strongly influences both the potential as well as the current density of the chloroform reduction peak, mainly due to the presence of OH^−^ in an alkaline Na_2_HPO_4_ solution, which is a specifically interacting anion. Moreover, the highest sensitivity of the Ag electrode toward CHCl_3_ reduction was observed in a neutral NaClO_4_ aqueous solution. It was found that the electroreduction of chloroform at the silver surface occurs via a concerted mechanism regardless of the type of the studied supporting electrolyte.

## 1. Introduction

Chloroform and other trihalomethanes (THMs) are known to be environmental pollutants [1], toxic, and possibly carcinogenic agents to humans [2]. The use of chloroform in consumer products (surgical anesthetic, cough syrups, toothpaste, etc.) was banned in the USA in 1976, as it was found to be carcinogenic in animal tests [3]. The issue of water contamination with THMs is reflected by the EU [4] and USA [5] regulations with the total trihalomethanes level in drinking water at maximum allowable averages of 100 and 80 μg L^−1^, respectively. The known sources of chloroform pollution are either environmental (volcanic emission and marine algae) or anthropogenic [6]. The CHCl_3_ can be formed during waste incineration or as a water chlorination by-product. Moreover, chloroform is still widely used as a solvent in the pharmaceutical industry, dyes and pesticides synthesis, fire extinguishers, rubber industry, etc. and, therefore, it can be emitted to the environment in a form of exhaust gas and wastewater.

Catalytic cathodic reduction at silver electrodes, aiming to convert organic halides to the corresponding de-halogenated hydrocarbons, has been reported as a promising approach toward the reduced abatement of such environmental pollutants [7]. At this point, most of the reported studies on the electrocatalytic reduction of organic halides were oriented to explore the mechanism of bond-braking reactions in nonaqueous solutions [8,9,10]. The aqueous solutions were also studied, regardless of the fact that the electrochemical reduction of many organic halides takes place at very negative potentials, at which a simultaneous hydrogen evolution reaction (HER) occurs, significantly increasing the energy consumption of the process [11,12,13,14,15,16,17,18,19]. Nonetheless, further studies on principal aspects of the chloroform electrocatalytic reduction at silver electrodes in aqueous solutions is a crucial step toward environmental applications.

To date, Rondinini et al. [20] studied the effect of the supporting electrolyte on the electrocatalytic activity of silver in nonaqueous solutions. They explored a large set of alkylammonium salts, and showed that the reduction potentials were spread widely in a range of about 300 mV. Authors reported that the major factor determining the reduction potential is the nature of the anion, while the cations exert a finer modulating effect. Moreover, the current density of the peak was also dependent on the supporting electrolyte. Fiori et al. [8] and Rondinini et al. [21] later confirmed the significant influence of the supporting electrolyte on the reduction potential of organic halides at Ag electrodes in a nonaqueous environment. For the chloroform reduction at silver electrodes in aqueous solutions, tetraethylammonium benzoate [22], KClO_4_ [14,17,23,24], NaClO_4_ [12], and NaOH [16] were reported as supporting electrolytes. NaClO_4_ and KClO_4_ are reportedly used due to negligible specific interactions of silver with perchlorate anions and sodium/potassium cations [25]. Taking into consideration the effect of the supporting electrolyte clearly demonstrated for nonaqueous solutions, it is surprising that no comparative study was reported to date for aqueous solutions. Therefore, in this work, aqueous NaClO_4_, NaH_2_PO_4_, and Na_2_HPO_4_ solutions were selected and studied as supporting electrolytes for the electrochemical reduction of chloroform at Ag.

## 2. Results and Discussion

During the reduction of chloroform at silver electrodes in nonaqueous solvents, 2 or 3 reduction peaks are observed [8,9,10,21,26], which can be ascribed to a gradual dehalogenation of the molecule, i.e., successive removal of halogen atoms [9]. In aqueous solutions, typically one wide peak of chloroform reduction, shifted toward less negative potential values as compared to nonaqueous solvents (up to 0.44 V), was observed [27]. The shift of the chloroform reduction peak toward less negative values, as compared to nonaqueous solvents, was attributed to the ability of water to enhance the turnover frequency of catalytic sites, as it favors the desorption processes [13]. It is also assumed that the electrocatalytic reduction of CHCl_3_ in an aqueous solvent is a complex phenomenon. Consequently, a superimposition of different peaks, corresponding to subsequent steps in the reaction mechanism, occurs [23], hindering the correct analysis of the process. Table 1 shows chloroform reduction peak potentials (E_p_), scan rates (ν), and concentrations of CHCl_3_ (c) reported in the literature.

**Figure 1:**
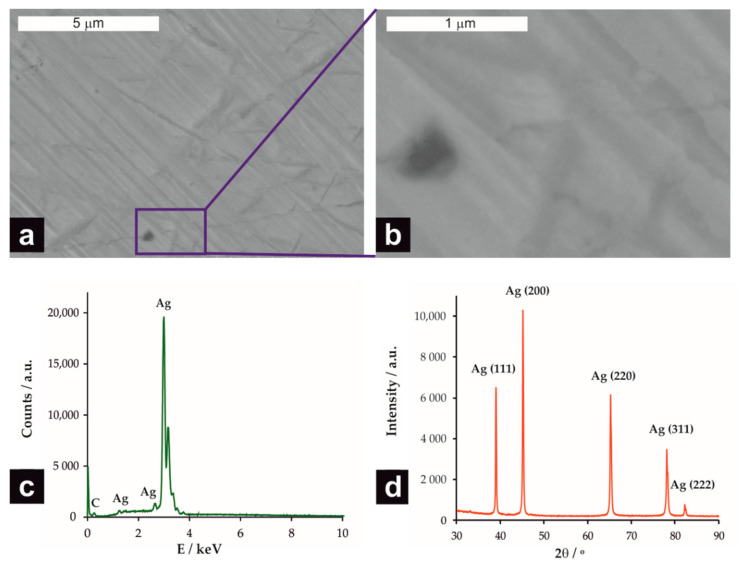
FE-SEM micrographs of the silver electrode (**a**) and a higher magnification of the selected area (**b**). The EDS analysis (**c**) and XRD diffraction pattern (**d**) of the Ag electrode.

From the data gathered in Table 1, one can conclude that the direct comparison of the influence of different electrode materials from the literature reports is not possible, as the scan rate, concentration of chloroform, as well as type and concentration of the supporting electrolyte, strongly differ. It was previously shown in cyclic voltammetry (CV) measurements that both the potential and current density of the chloroform reduction peak strongly depend on the concentration of CHCl_3_ [17]. The CV measurements were performed at the silver electrode (Figure 1) in order to assess the effect of the supporting electrolyte on the potential and current density of the chloroform reduction peak. As expected, the EDS spectrum of the Ag electrode (Figure 1c) confirmed its chemical composition and, consequently, the planes (111), (200), (220), (1311), and (222) indexed to face-centered-cubic silver (JCPDS No. 04-0783) were detected in the XRD pattern.

**Figure 2:**
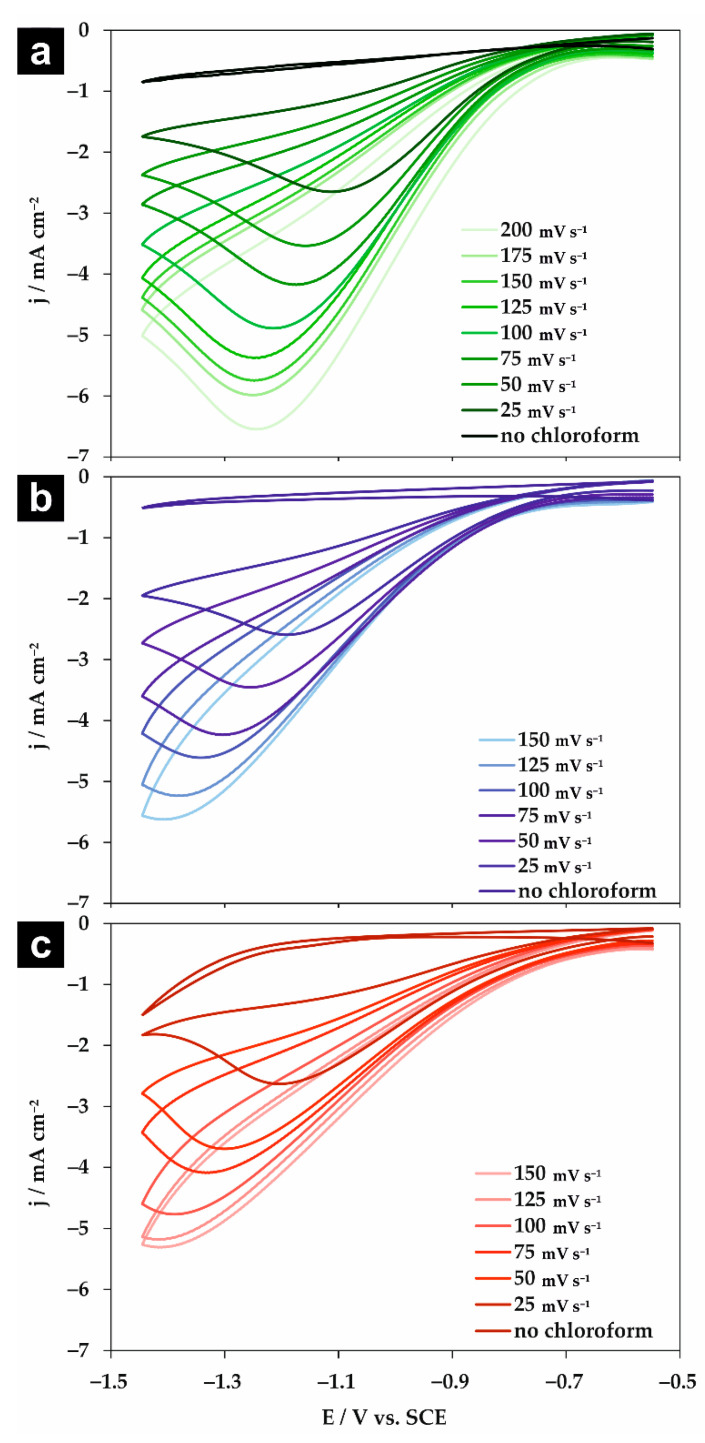
Cyclic voltammograms obtained at the silver electrode in the absence and presence of 8.26 mM chloroform in 0.05 M (**a**) Na_2_HPO_4_, (**b**) NaClO_4_, and (**c**) NaH_2_PO_4_ solutions for selected scan rates.

The voltammograms obtained at the Ag electrode in the absence and presence of 8.26 mM chloroform for selected scan rates are shown in Figure 2 for 0.05 M Na_2_HPO_4_, NaClO_4_, and NaH_2_PO_4_ solutions. The potentials are referenced to the saturated calomel electrode (SCE). In all studied electrolytes in the potential range of −0.547 to −1.447 V vs. SCE, a competitive reaction (i.e., decomposition of water or supporting electrolyte) was not observed. Only small capacitive currents were observed in the absence of chloroform. Conversely, independently of the studied electrolyte, after the addition of chloroform, one irreversible peak was observed similarly to previous reports (Table 1). Both the potential as well as the current density of the chloroform reduction peak shifted with the scan rate. Such an effect was previously reported for the chloroform reduction in aqueous solutions at Ag [17] and during benzyl chloride reduction in acetonitrile at Ag [29] and AgPd [30] electrodes. It is important to note that the magnitude of the shift strongly depends on the type of studied supporting electrolyte—especially when the results are compared with previously reported data for the chloroform reduction in 0.05 M KClO_4_ in the same setup [17]. The most significant shift in the reduction potential and highest current density were observed for Na_2_HPO_4_. This fact will be discussed later.

**Figure 3:**
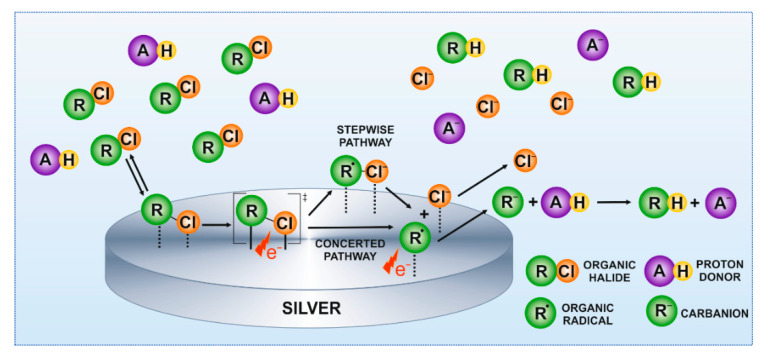
Schematic representation of the mechanism of chloroform reduction at the surface of Ag electrode in aqueous solutions.

The reduction of organic halides occurs according to a stepwise or concerted pathway (Figure 3).

In the stepwise mechanism, an intermediate radical anion is formed before the C–X bond cleavage. In the concerted mechanism, the electron transfer and the C–X bond cleavage occur simultaneously. Those reactions are considered the slowest step of the reduction. The second electron transfer to the formed radical leads to the formation of organic anions, and is recognized as a very fast step occurring at less negative potentials. The created carbanion is protonated rapidly by a proton donor [27]. The cleavage mechanism of organic halide R–X bonds depends mainly on the type of reduced molecule and electrode material [31]. A kinetic indicator κ can be a useful tool to discriminate between the mechanisms involved in the R–X bond cleavage [32], and can be determined by voltammetric analysis using Equations (1) and (2) [26]:

(1) $$\kappa_{1}=-\frac{1.151\mathrm{RT}}{F\left(\frac{\partial E_{p}}{\partial \log\nu }\right)}$$

(2) $$\kappa_{2}=-\frac{1.857\mathrm{RT}}{F\left(E_{p/2}-E_{p}\right)}$$

where: κ—electron transfer coefficient; R—universal gas constant, R = 8.3145 J mol^−1^ K^−1^; T—temperature, K; ν—scan rate, V s^−1^; E_p_—reduction potential of chloroform, V; E_p/2_—potential corresponding to half of the peak current, V; F—Faraday constant, F = 9.6485 · 10^4^ C mol^−1^.

**Figure 4:**
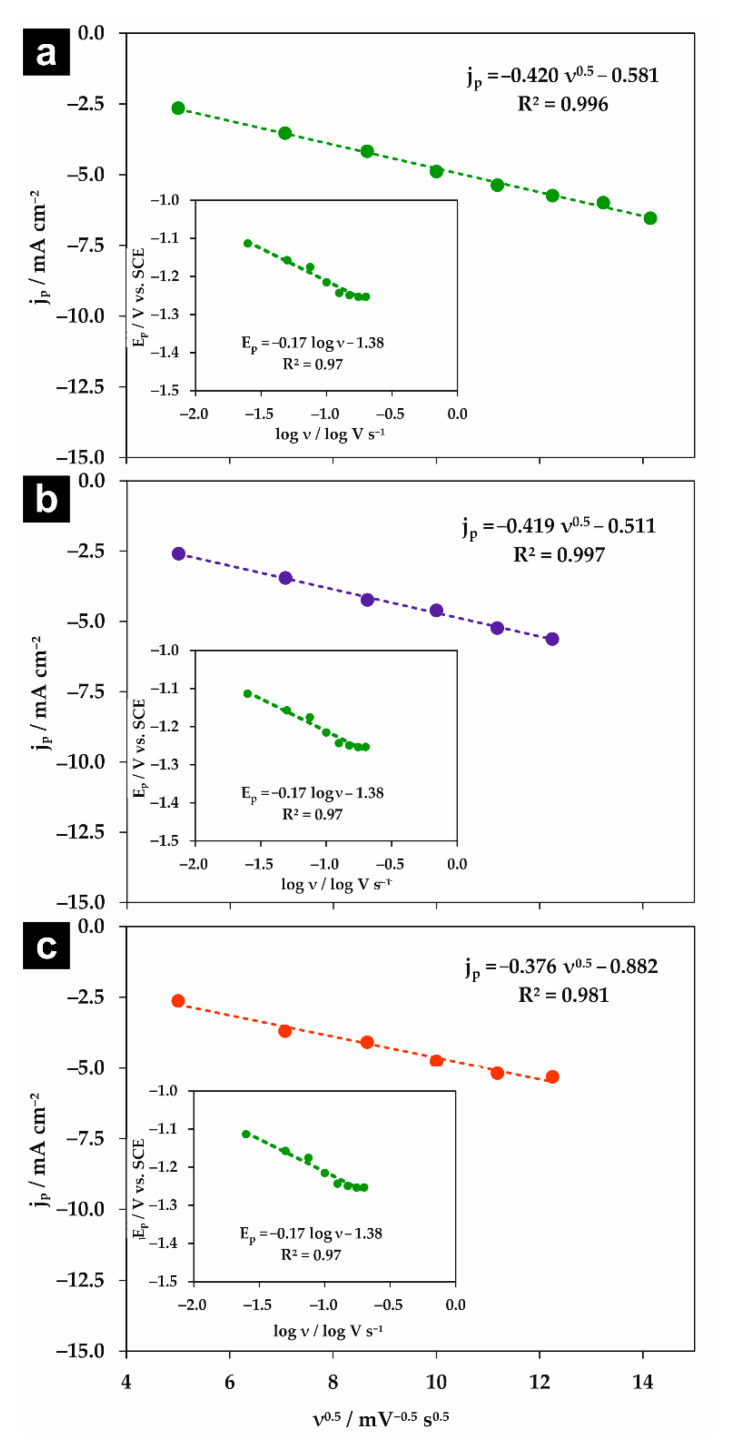
The dependencies of the cathodic current density of the CHCl_3_ reduction peak (j_p_) vs. the square root of the scan rate (ν^0.5^) measured in 0.05 M (**a**) Na_2_HPO_4_, (**b**) NaClO_4_, and (**c**) NaH_2_PO_4_. Insets: The corresponding dependencies of the peak potential (E_p_) vs. log ν.

If κ is in the range between 0.35 and 0.5, the process occurs via the stepwise mechanism with an electrochemical rate-determining step. For κ < 0.35, the reaction follows the concerted mechanism and κ values can be considered as a transfer coefficient [17]. Moreover, Equation (1) clearly shows that the observed shift in the reduction potential with scan rate is correlated with the electron transfer coefficient. As can be seen in Figure 4, the peak current density (j_p_) is linearly dependent on the square root of the scan rate (ν^0.5^) for all used supporting electrolytes, which indicates that the reaction is diffusion-controlled [31]. Since E_p_ vs. log ν dependencies (insets in Figure 4) are linear with negative slopes, it was possible to estimate κ values (Table 2) from Equations (1) and (2).

**Figure 5:**
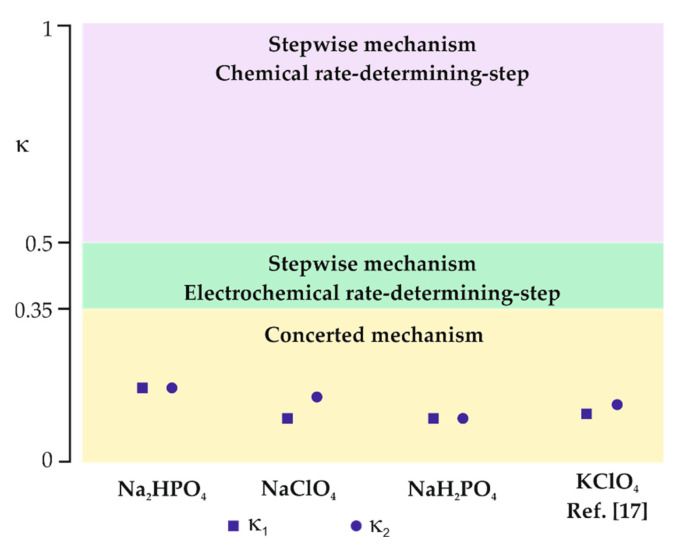
Kinetic indicator κ for identification of the mechanism involved in the R-X bond cleavage together with experimental κ values obtained for the reduction of chloroform in different aqueous supporting electrolytes.

For all studied supporting electrolytes, the observed current density of the chloroform reduction peak is similar; however, the peak potential strongly differs. Moreover, the calculated κ values are significantly lower than 0.35 and are in good agreement for both estimation methods. It indicates that the chloroform reduction in studied supporting electrolytes takes place via the concerted mechanism at the surface of the polycrystalline silver electrode (Figure 5). Such a mechanism is often observed for cases when a proton donor is present in the solution [31]. For instance, the transfer coefficient reported for the chloroform reduction at the Ag electrode was in the range of 0.11–0.13 for 8.26 mM CHCl_3_ in a 0.05 M aqueous solution of KClO_4_ [17]. The charge transfer coefficient depends on the peak potential of analyte reduction [33,34]; therefore, observed discrepancies are expected.

Similarly, as in the case of the electrocatalytic reduction of organic halides in nonaqueous solutions, the selected aqueous supporting electrolytes influence the reduction potential of the studied compound [20]. Importantly, the nucleophilic character of the hydrogen phosphate ion (HPO_4_^2–^) has to be considered, which is about twice as great as for the hydroxyl ion, leading to hydrolysis-like reactions and providing easier removal of Cl^−^ ions [35]. This could lead to *(i)* lowering of the concentration of chloroform in the electrolyte and, therefore, a less negative potential of the chloroform reduction, and *(ii)* a slightly higher cathodic current density of the reduction peak due to a higher turnover frequency in the case of Na_2_HPO_4_. However, further extensive studies are necessary for the better understanding of this phenomenon. Moreover, in the aqueous solutions, additional hydrolysis of chloroform can occur, according to a neutral mechanism (Equation (3)) or an alkaline-catalyzed mechanism (Equation (4)) [35]:

CHCl_3_ + 5H_2_O → HCOOH + 3H_3_O^+^ + 3Cl^−^ (3)

CHCl_3_ + 4OH^−^ → HCOO^−^ + 2H_2_O + 3Cl^−^ (4)

The neutral hydrolysis is the dominant pathway at pH < 4. Between pH 4 and 8, none of the hydrolysis mechanisms are predominant, and at pH > 8, the alkaline-catalyzed hydrolysis pathway becomes important due to the strong neutrophilic character of the hydroxyl ion [35]. The presence of OH^−^, specifically interacting ion in a solution, is therefore another reason for the less negative potential of chloroform reduction observed in an alkaline solution due to a lower concentration of chloroform. The hydrolysis half-life of chloroform at room temperature is 1000 years at pH = 7, and even longer for more acidic solutions; therefore, the influence of hydrolysis could be neglected.

**Figure 6:**
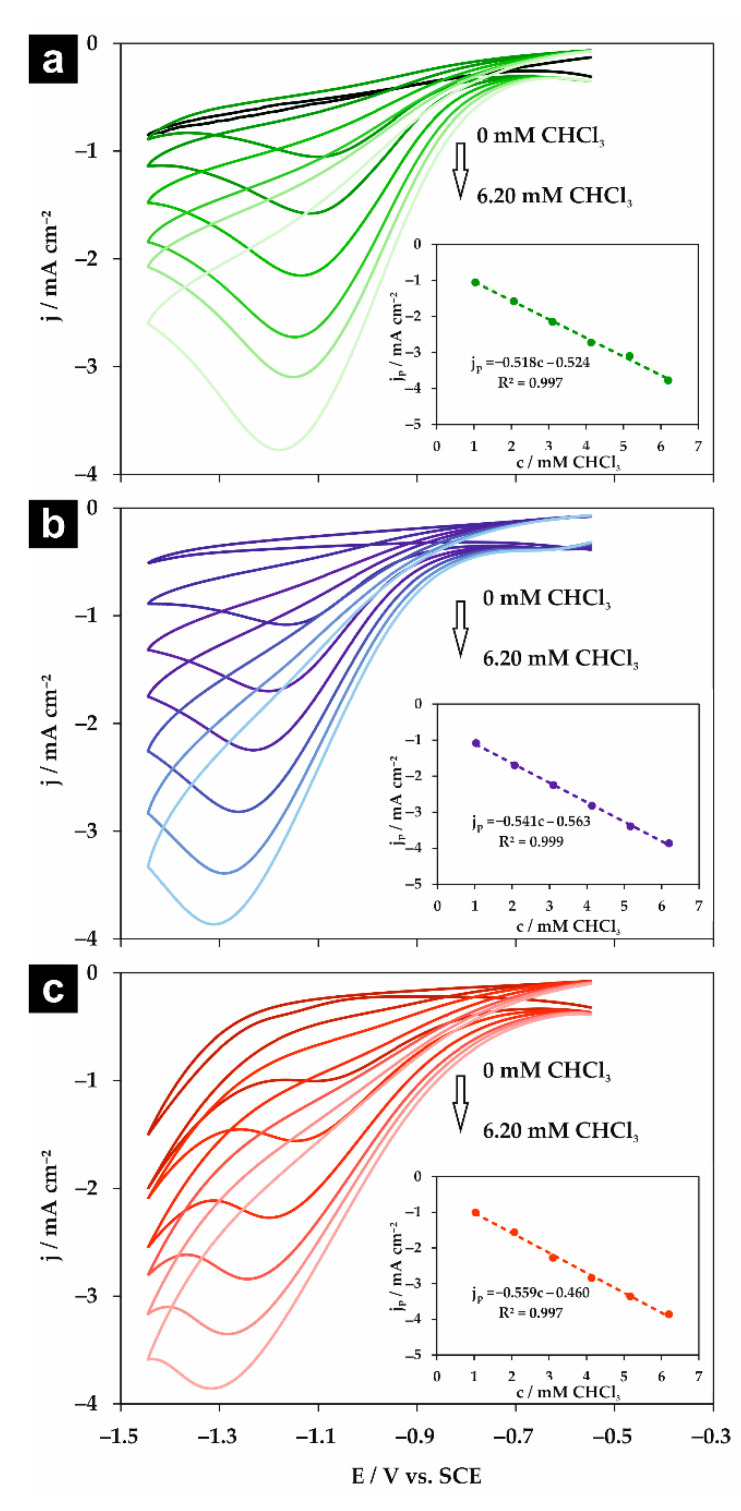
Cyclic voltammograms obtained at 100 mV s^−1^ for the silver electrode in a 0.05 M solution of (**a**) Na_2_HPO_4_, (**b**) NaClO_4_, and (**c**) NaH_2_PO_4_ containing trichloromethane in the concentration range of 0–6.20 mM. Insets: The corresponding dependencies of the peak cathodic current density of CHCl_3_ reduction (j_p_) vs. chloroform concentration.

Subsequently, the influence of chloroform concentration on the peak potential and current density for all studied electrolytes was explored. The CV measurements were performed in a 0.05 M solution of supporting electrolyte at the scan rate of 100 mV s^−1^. The registered voltammograms are shown in Figure 6. For all studied supporting electrolytes, a linear correlation between the peak current density and CHCl_3_ concentration was observed for the concentration range of 1.03–6.20 mM (see insets in Figure 6). Similarly to previous reports [10,17], the potential of chloroform reduction shifts toward more negative values with its concentration. Therefore, it is important to consider the concentration of organic halides, when the catalytic activity of different electrodes is compared. The calculated sensitivity, limit of detection (LOD, calculated as 3σ_b_/a), and limit of quantification (LOQ, calculated as 10σ_b_/a) are gathered in Table 3. Clearly, the lowest LOD and LOQ values, as well as the highest sensitivity, were obtained for NaClO_4_, which implies an inert character of the supporting electrolyte and justifies the claim that this supporting electrolyte is the best choice among those studied here.

## 3. Materials and Methods

Pure disodium phosphate (Na_2_HPO_4_·2H_2_O), sodium phosphate (NaH_2_PO_4_·H_2_O), and sodium perchlorate (NaClO_4_·H_2_O) were purchased from Sigma-Aldrich (St. Louis, MO, USA), and chloroform (CHCl_3_) was acquired from Chempur^®^ (Piekary Śląskie, Poland). The polishing Al_2_O_3_ fine abrasive powder was purchased from Alfa Aesar^®^ (Thermo Fisher Scientific, Lancashire, United Kingdom). Highly deionized water (18.2 MΩcm) was obtained using the MilliQ^®^ Millipore System (Merck KGaA, Darmstadt, Germany).

All electrochemical measurements were performed in a custom-made, conventional three-electrode setup with the Gamry Instrument Reference 3000 potentiostat/galvanostat (Warminster, PA, USA). For cyclic voltammetry measurements, Teflon-coated polycrystalline silver (Ag, 7 mm diameter, 99.999% Specpure, Johnson Matthey Chemicals Ltd., England) served as a working electrode, the Ag|AgCl|Cl^−^ (3 M NaCl) electrode in a Luggin capillary (G0100 Vycor, BASi, West Lafayette, IN, USA) filled with a 0.1 M solution of the selected supporting electrolyte was used as a reference electrode, and a Pt wire coiled on a Teflon^®^ frame was used as a counter electrode [17]. The CV measurements were recorded at the potential range from −0.547 to −1.447 V at selected scan rates (25–200 mV s^−1^) and chloroform concentrations (0–6.02 mM). All used electrolytes, i.e., NaClO_4_, NaH_2_PO4, Na_2_HPO_4_, were prepared using deionized water. Before each series of experiments, the surface of the silver electrode was polished with the Al_2_O_3_ powder, and electrochemically cleaned between each measurement in the 0.05 M supporting electrolyte by CV cycling in abovementioned potential range. Due to the fact that in the literature, the reduction potentials for organic halides are consistently provided versus the SCE, all potentials measured in this study were also recalculated to this reference.

The surface morphology of Ag electrode and its composition was studied using a field emission scanning electron microscope (FE-SEM, Hitachi S-4700, Hitachi High-Tech Corporation, Tokyo, Japan) equipped with an energy-dispersive X-ray spectrometer (EDS, Noran System 7, Thermo Electron Scientific Instruments LLC, Madison, WI, USA). The crystallinity of the Ag electrode was examined using a Rigaku Mini Flex II diffractometer with Cu K α radiation (1.54060 Å) at 30–90° 2θ with a step size of 0.005° and at a rate of 5° min.^−1^.

## 4. Conclusions

In this work, for the first time, the influence of the supporting electrolyte on the potential of chloroform reduction in aqueous environment at the surface of the silver electrode was reported. Similarly to previously reported nonaqueous supporting electrolytes, we observed that both the peak current density and peak potential of the chloroform reduction depend on the type of the used supporting electrolyte. On the other hand, it was found that the electroreduction of chloroform at the silver surface occurs via a concerted mechanism regardless of the type of studied supporting electrolyte. The highest sensitivity of the Ag electrode toward CHCl_3_ reduction was observed in a neutral NaClO_4_ aqueous solution. NaClO_4_ is widely reported in the literature as an inert aqueous supporting electrolyte for the electrocatalytic reduction of organic halides at Ag. However, the influence of other supporting electrolytes and noninert additives affecting the solution pH is often ignored. This is particularly important for the electrocatalytic reduction of organic halides in real water samples, where multiple anions and cations can be present.

## Figures and Tables

**Table 1:** Overview of experimental data for the chloroform reduction in aqueous solutions.

Electrode	E_p_, V vs. SCE	ν, V s^−1^	_C_ CHCl_3_, mM	Supporting Electrolyte	Ref.
Ag	−1.05	0.1	5.0	0.010 M TEAB	[22]
Ag	−1.0	0.1	5	0.035 M NaClO_4_	[28]
Ag	−1.32	0.1	8.26	0.05 M KClO_4_	[17]
Ag NWs	−1.15	0.1	8.26	0.05 M KClO_4_	[17]
Ag NPs @ modified carbon powder	−0.85	0.2	10	0.1 M KClO_4_	[15]
Ag NPs in C-ME	−0.6–−1	0.3	10	0.1 M KClO_4_	[14]
Ag	−1.127	0.1	10.63	0.1 M NaOH	[16]
Ag NCs	−1.067	0.1	10.63	0.1 M NaOH	[16]
Ag NWs	−0.957	0.1	10.63	0.1 M NaOH	[16]

TEAB—tetraethylammonium benzoate, NWs—nanowires, NPs—nanoparticles, C-ME—microcavity electrode, NCs—nanocones.

**Table 2:** Data for the chloroform reduction at the silver electrode in selected aqueous supporting electrolytes.

Electrolyte	pH	E_p_, V vs. SCE at 100 mV s^−1^	j_p_, mA cm^−2^ at 100 mV s^−1^	$\frac{\partial E_{p}}{\partial \mathrm{logv}}$	κ_1_	κ_2_
Na_2_HPO_4_	9.4	−1.215	−4.89	−0.171	0.17	0.17
NaClO_4_	7.0	−1.344	−4.61	−0.284	0.10	0.15
NaH_2_PO_4_	4.6	−1.389	−4.77	−0.287	0.10	0.10

**Table 3:** The analytical parameters of the chloroform reduction at silver electrode in different aqueous solutions.

Electrolyte	LOD, mM	LOQ, mM	Sensitivity, mA cm^−2^ M^−1^
Na_2_HPO_4_	0.348	1.16	1.04
NaClO_4_	0.204	0.68	1.10
NaH_2_PO_4_	0.354	1.18	1.02

## Data Availability

Data sharing not applicable.

## Sample Availability

Samples of the compounds are not available from the authors.
